# On Pulmonary Consumption, and on Bronchial and Laryngeal Disease, &c.

**Published:** 1847-10-01

**Authors:** 


I. On Pulmonary Consumption, and on Bronchial and Laryngeal Disease, with Remarks on Places of Residence chiefly resorted to by the Consumptive Invalid. By Sir *Charles Scudamore*, M.D., F.R.S., &c. &c. 8vo, pp. 259. London, Churchill, 1847.

II. Cold and Consumption; or, Consumption, its Prevention and Cure by Cold as a Constitutional, and Inhalation as a Local, Agent, &c. &c. By *Henry C. Deshon*, Member of the Royal Colleges of Physicians and Surgeons of London, &c. 8vo, pp. 153. London, Renshaw, 1847.

It is now ten or twelve years—it may be more—since Sir Charles Scudamore published a work on the treatment of Phthisis by the inhalation of Iodine. It was not well received by the profession at the time, in consequence of an air of mystery that was cast around the use of the remedy, in a manner which was rightly deemed not altogether consistent with the usage of an honourable profession. Sir Charles thereupon promptly made public his mode of practice; but we have never understood that it has been used with any satisfactory results by other medical men to warrant its general adoption, and it may therefore be fairly presumed that it, like too many other vaunted remedies for pulmonary consumption, has been found inefficacious, if not positively hurtful. We have seen it tried under Sir Charles' own superintendance; but the results of the trial most assuredly did not encourage us to repeat it in our own practice.

It may be as well here to specify Sir Charles' formula, and the directions he gives for using it.

℞. Iodinii puri,
Potasii iodid. āā gr. vj.
Aquæ destillat. ℥v. ʒvj.
Alcoholis ʒij. M. fiat mistura, in inhalationem adhibenda.

"But invariably I direct the addition of a *saturated* tincture of the dried leaves of conium, which in the most favorable manner softens the action of the iodine solution, and tends to soothe the bronchial mucous membrane. Of the iodine solution I commence with the dose of 30 minims, and increase it by 5 or 10 at a time, in a gradual manner, according to its effects and the nature of the case, till I may perhaps carry it to 240 minims; but, in the majority of instances, I confine my range to 180 minims. Whatever may be the quantity I use for each inhalation, I constantly direct that two-thirds of it be put at first, the other third when half of the time for inhaling has expired; otherwise it would be too strong at first and too weak at last; 30 minims of the tincture is the ordinary dose which I prescribe, and this need not be divided, nor does it in general require increase, as it is so much less volatile than the iodine, and enough of strength remains; but, if much of its soothing influence be wanted, either to allay irritable cough, or to act as a soporific, a drachm, or even a drachm and a half, may be employed; but in such case, it is better to use it in divided portions. The water, to which these preparations are to be added, should be of a temperature of from 115° to 125° Farenheit;—as a medium, 120°; a little more or less is not material. The whole should be well blended by shaking the inhaler. This should be constructed of glass, for a metallic one instantly decomposes the iodine. Its tubes should be capacious, and the inhaler should never be quite half-filled; for if these two last circumstances are not carefully attended to, much inconvenience would arise, the inhaling would be rendered difficult, and which by proper attention is so perfectly easy a process, that an invalid with the weakest respiratory powers does not experience any difficulty.

"The last part of these preparatory steps, for the purpose of keeping up the proper temperature of the contents of the inhaler, is to place it in an open vessel, large enough to allow of the inhaler being a little removed from its sides. Water, of a temperature from 120° to 130°, is to be put into it, enough to rise to about two-thirds of the inhaler; and, to prevent any inconvenience from the vapour which issues, the vessel should be covered over with a piece of thick paste-board, neatly fitted.

"Now desire the patient to inhale by making a rather deep inspiration, then to relax, or take off, the lips from the mouth-piece; and to inhale again immediately, carrying on the process effectively, so that the medicated vapour shall pass into the deep air-passages, but not in a quick and fatiguing manner. At the first time of using it, five or six minutes will be sufficient; but in the progress of the treatment it may be extended to twenty, twenty-five, or thirty; but I seldom in my direction exceed twenty. The frequency of repetition is from twice to thrice in the day; commonly thrice, for the first four or six weeks." P. 110.

Such is the remedy, from the use of which Sir Charles assures us that he has often derived the most surprising and truly gratifying results in the treatment of even aggravated cases of tubercular Phthisis. He does not indeed trust to it alone; but it is abundantly obvious that the other remedies employed are regarded by him but as adjuvants, and that it is to this one in particular that the credit of his succsss is believed to belong. As might be expected, he gives minute details of some of the wonderful cures that he has effected. The question is, can we place sufficient reliance upon Sir Charles' diagnostic skill to warrant us in recognising the curative efficacy of his practice? It is most unfortunate that he has not adduced any cases from the experience of other medical men, or, at all events, where their concurrent testimony might have been had, in confirmation of his own very favourable statements. In Case VIII., indeed, we learn that another medical gentleman was in attendance; but, alas! his testimony is one of the most damaging things in the world to the credit of our author's sagacity; for the ignorance displayed by this witness of the subject on which he gives his opinion is so transparent, that one might almost suspect that he is not an educated member of the profession. What will our readers say of the information contained in the following passage?

"The left lung gave strong puerperal resonance; the right lung was pectoriloquous, from the root to the mamma; there was much gurgling, and percussion was dull over the greater part of the right side; it was also much smaller than the left, and hollow at the clavicle. The body was much reduced; he had profuse hectic sweats, and the expectoration was copious, puriform, and very offensive; the pulse rapid. His debility was so great, that, to use his own words, he felt to be dying from day to day. The night perspirations were most profuse, and he was often sleepless. On the first inhalation, he expressed himself very sensibly relieved; afterwards, his breathing was never oppressed in going up stairs, as it was before using the inhalation; and, with a little more interval, he was able to walk two miles without fatigue." P. 140.

The report of the case, to which these remarks apply, may be fairly taken as a specimen of the evidence which Sir Charles adduces in favour of *his* method of treatment. It is headed—"*Tubercular Phthisis; the existence of a large cavity unquestionable. Symptoms of the case highly alarming; recovery, which lasted for a long period.*" The patient was believed to be in a most confirmed consumption; the right lung being supposed to contain a large tuberculous excavation, and the left one to be infiltrated with tubercles in a crude state. Great emaciation, afternoon hectic, and night sweats, had existed for a considerable time. Now for the treatment that was adopted, and its declared results:

"The patient inhaled the mixture of iodine and conium regularly three times a day, at first for ten minutes, afterwards gradually increased to twenty; small blisters were applied to the chest from time to time; the lotion of tannin infusion, with acetic acid and eau de Cologne, was applied night and morning to the skin, followed by the use of the flesh-brush. Internally, pills composed of pilula. hydrarg. camphor. and c. colocynth. extract, were given at night, occasionally followed by a morning aperient draught; a strong infusion of the cortical part of sarsaparilla, with alkali and gentian, was used twice in the day; and, to procure comfortable sleep at night, he took a soothing morphine syrup, acidulated with diluted sulphuric acid. The plan of diet was changed to one highly nutritious; and such were the languor and debility, that wine, the best port and sherry, was allowed with more than usual freedom. He usually took three or four glasses in the course of the day, in addition to a pint of sound draught porter, not only without disagreement, but with every sense of benefit. He had sometimes alarming attacks of exhaustion, at the commencement of my attendance. His diet had been too much restricted, and he had indeed said that he was 'dying from starvation.' After a few weeks, iron and quinine were administered in conjunction, instead of the other medicines." P. 140.

Within three weeks, "the specific symptoms were most materially relieved;" and in three weeks more, he was on the high road to recovery. Sir Charles saw his patient two or three months afterwards, and then he found "a remarkable diminution in the extent of the pectoriloquism, with an evident amelioration in the condition of each lung. The râles had ceased; and by auscultation there was satisfactory evidence of a very improved respiration. The expectoration continued, but was much lessened in quantity, and almost free from its former offensive odour. It appeared to me that the tuberculous cavity was in a favourable progress of healing; and certainly the whole aspect of the patient was promising a fair recovery; for to regain perfect health could not be expected, when so much disorganisation of lungs had been produced, existing in conjunction with an unhealthy liver." It would appear that this patient recovered his health and strength so much that he was able to return to the West Indies, where he remained for nearly a twelvemonth, that he then returned to this country, "passed the winter in a cold wet part of the North of Scotland, and there took cold very seriously, and had an attack of acute symptoms which terminated fatally."

All that we shall say respecting this case is, that we feel it our duty to caution our less experienced brethren against expecting any thing at all like the success which Sir Charles considers to have been the result of his method of treatment; else they will assuredly be most grievously disappointed.

But it is not only in aggravated cases of tuberculous disease of the lungs that Sir Charles has succeeded. Even when there has been Empyema existing at the same time, he has met with the most gratifying results. Case XX. is one in point, provided indeed the reader is satisfied that there was any purulent effusion into the chest at all; for certainly the most characteristic symptoms of this very serious complication were not present, if we may judge from the report. The reader may determine for himself from the following narrative of the symptoms: there was an open abscess, we should remark, between the 5th and 6th ribs on the left side.

"The fifth, sixth and seventh ribs were elevated, giving to the side a very swollen appearance. The orifice of the abscess had its edges quite inverted. There was a slight purulent discharge; and it was remarkable that this alternated with the expectoration: when the one was free, the other was very slight. Of this fact I was several times an eye-witness. By auscultation and percussion, the following evidence was afforded: considerable resonance in the upper part of the right lung, and still more remarkable in the right axilla. Imperfect respiration in the upper part of the left lung; and below also it was imperfect and more indistinct.

"Sound duller than natural on the right side; dull at the inferior part of the left, especially when the patient was in the erect position; and becoming clearer when he lay on the opposite side. I drew the inference that there were tubercles in the upper part of each lung, but particularly the right; that there was effusion into the cavity of the left pleura; that nature had performed the operation for empyema in producing the external abscess; and that, internally, a communication had been formed, by ulceration, between the bronchi and pleura." P. 169.

In this case, too, the inhalation of *iodine* and *conium*, in conjunction with a generous diet and the use of sarsaparilla and other alteratives, effected the most satisfactory amendment: "indeed, it much exceeded all expectation." This patient too, like the preceding one, after having made such wonderful progress under Sir Charles' treatment, very imprudently exposed himself to wet and cold, which brought on a recurrence of all the bad symptoms and proved fatal. Unfortunately, there seems never to have been any examination after death of the state of the thoracic viscera; so that we are left in doubt as to the correctness of the diagnosis that had been formed.

What tends very greatly to diminish our confidence in the full accuracy of our author's statements in reference to the surprising cures which he has effected, is the circumstance of his, every now and then, seeking to disarm incredulity by protestations of his candour and love of truth. For example, in one passage he says,—"I can conscientiously declare that I have not been guilty of the least exaggeration in any part of my narrative of cases." Such language sounds to us very much like that of one who deprecates the charge of being a story-teller. Why should any man, assured of the perfect truthfulness of his statements, exhibit such a timorous cringing spirit? Truth is ever bold, nor seeks to win favour by assuming an air of obsequious humility. Besides, we like not such remarks as these:—"If I had made my own credit and reputation a selfish consideration in the class of cases to be treated by iodine inhalation, I should have rejected all such as were evidently in their nature so confirmed and desperate as to preclude all chance of recovery; but as inhalation has the property of mitigating symptoms, where it cannot accomplish more, I have always felt it my duty to employ it in phthisis, if requested by the patient, even under most unfavourable circumstances of the disease;" and again—" If I had been governed by a rigid solicitude for the credit of inhalation, I might have declined the application of the treatment to such a case as this, at the first view so evidently hopeless." Who ever suspects an honest physician of any other motive save the simple one of seeking to relieve distress and sufferings of his patient? Away then with all such silly, and worse than silly, talk about his credit and fame. Neither do we understand what our author means when he tells us that " no one is more fully aware than himself of the doubtful balance in which a physician places his feelings and his reputation, who engages in the treatment of consumption." The balance need never be doubtful either one way or the other, provided, all the while, he knows that nought save the dictates of holy truth and humanity has been the actuating principle of his conduct.

In another respect, the language employed by Sir Charles is not very likely to conciliate the approbation of the profession. The following passages will best serve to illustrate what we allude to.

" I adopt the opinion that tubercular phthisis is a specific blood disease, that there exists a tuberculous condition of the blood, and which, in the strongly marked examples of hereditary phthisis, is born with the individual as a germ." P. 55.

" The tubercular virus may exist in different degrees of intensity in different persons; in one individual, leading to slight indications of consumption, what is called a tendency to it; in a second subject, to the disease well-marked, in the chronic form; in a third, the acute disease, running a most rapid course." P. 57.

" In conclusion, although the method of practice which I have advocated in these pages is the most useful and the most likely to succeed in tubercular phthisis of any with which I am acquainted, it falls short of that which, for the good of humanity, I desire. The *desideratum*, as I conceive, is a medicine which shall be found to exert a successful *specific* agency against the tuberculous poison; to neutralise it, and effect a radical cure!" P. 216.

One can scarcely believe that any person, who talks of a *specific* for the tuberculous poison, can have very accurate notions of pulmonary consumption. It may be as well to mention here that Sir Charles seems to attribute the therapeutic agency of his remedy rather to its alterative effects upon the system in general, than upon its local operation upon the diseased tissue to which it is applied. The only evidence, however, adduced in favour of this idea is contained in the following paragraph.

" That the introduction of iodine into the system through the medium of the lungs is effected, I have had the proof occasionally by witnessing some of those inconvenient effects on the constitution which its use as an internal medicine, or as rubbed in the form of ointment on the thyroid gland, now and then produce. But I am happy to add, that such instances are so very rare, as not to form any objection to its employment. I am convinced that it does not happen so much as once in fifty times. The disagreement in question is a peculiar nervousness, a tremor, and a timidity; but no disorder of the stomach or bowels; as is liable to happen from the ordinary taking of iodine." P. 211.

Before taking leave of Sir Charles, we wish to call the attention of the reader for a few minutes to some observations he has made, in the early part of his volume, on the question of the great increase of the animal heat in cases of Consumption.

"It is a curious pathological fact, as I have found in a very extensive examination to be verified without a single exception, that in every case of tubercular phthisis the animal heat is more or less raised beyond the healthy standard. This may be stated as a mean at 96·5. It is always found highest in the morning at the time of rising from bed, with all persons. In the course of the day it is influenced by certain circumstances, and is raised by exercise; particularly in the fresh air of the country. In phthisis, I have found it range from 99° to 105°. I consider that the examination of the animal heat assists our diagnosis as to the existence of tubercles. In a doubtful case, I am pleased to find the animal heat not higher than 98°.

"It would be foreign to my present purpose to enter into the interesting, beautiful, yet difficult subject of animal heat; or attempt the consideration how far this function is to be referred to chemical action taking place in the lungs, how far to vital influence, and how far to the nervous system; but I believe it is on all hands agreed that the most immediate and influential cause of the production of animal heat is the combustion of carbon, brought in the venous blood to the ramifying capillaries, in order to receive aeration and the all-important influence of oxygen introduced by the air-passages.

"In this chemical view of the subject, and to which I now confine myself, it appears, I think, surprising, when we consider how much of tuberculated lungs is excluded from the aerating process, either from the compression of the air-cells by the tubercles, or their actual occupation by these foreign bodies, that the animal heat, instead of being lower, as we might imagine, as a consequence of the surface for aeration of the blood being greatly lessened, is actually found to be higher than in the natural healthy state of the lungs." P. 30.

After relating some experinents which go to shew that the quantity of carbonic acid evolved during respiration is greater in a phthisical patient than in a healthy person, our author remarks :

"It appears to me that, in the relation of these experiments, I have offered strong evidence that in tubercular phthisis, notwithstanding the organic limitation of the function of the lungs, from the obstruction of the air-cells by tubercles, the important process of the decarbonisation of the blood, the combustion of carbon, and increased animal heat beyond the healthy standard, go on with more rapidity than in sound lungs. An increase of the animal heat does not ensue from merely quickened respiration, as is shown in the examples of running up to the top of a lofty building; nor even more than a degree in the paroxysm of spasmodic asthma. I consider, indeed, that in some cases of this kind, where almost asphyxia takes place, the reverse would happen, and the animal heat be found below the natural standard.

"There is in tuberculated lungs an increased activity of function; and hence the hectic fever, so urgent in acute phthisis, and the hectic irritation, although often hardly amounting to evident fever, in the chronic form of the disease. The actions of the animal economy, when unnaturally hurried, are not so healthily performed. We may suppose that the oxidation of the blood being effected in so rapid a manner, cannot be so favourably accomplished; and also a morbid excitement prevails in the whole system. The nervous system is morbidly sensitive. Although the appetite may be good, and abundance of food be taken, yet nutrition is imperfect, and the body wastes—a consequence much to be referred to the imperfect and unhealthy performance of assimilation and sanguification; at the same time that the absorbents generally are probably thrown into a state of morbid activity." P. 33.

The general result of Sir Charles' observations and experiments is, that, "when the lungs, or mucous membrane of the air-passages, are in a state of irritation from disease, the animal heat is always more or less raised beyond the natural standard." There can be no doubt but that a persistent increase of the animal heat, and (as always happens at the same time) of the pulse, is an almost infallible indication of an inward irritation; and that inward irritation is, in the majority of instances, dependent upon some pulmonic lesion.

The work of Dr. Deshon—who, we observe, lives at Budleigh Salterton in Devonshire—is altogether a most discreditable production, and one utterly unworthy of any educated and honourable member of our profession. We strongly suspected its character from its very title, and the suspicion was confirmed by the circumstance of our learning that no copy had been sent to the medical journals for review. But this attempt at latency will not preserve the author from exposure.

The first two chapters profess to give an anatomical and physiological description of the thorax and its viscera. We must find room for one or two *morçeaux* of the silly stuff of which they consist. For example, we are told that—

"The lungs and heart, conjointly, may not inaptly be regarded as a distillatory apparatus; whereof the lungs resemble the furnace, to which the blood, exposed in a convenient retort, the branches of the pulmonary artery, and purified of its grosser parts by due combustion, is returned by the pulmonary veins or worm, to the heart or receiving vessel, to be from thence conveyed by the principal artery of the body, or main pipe, and by its innumerable branches for universal distribution." P. 5.

"As a general rule, it may be said that in aquatic animals, the lungs are prolonged from within outwardly to meet the air with which the water is saturated; but in terrestrial beings, on the contrary, the lungs are contained in a cavity into which the air enters to meet the blood. The utility of this arrangement is obvious, namely, to present to the gill of the former, by constant change and motion, the largest possible amount of air; which, on the other hand, being exhibited to the latter in a more pure and undiluted state, every end is accomplished by a smaller quantity entering the pulmonic sac, or receiver. Again, it is absolutely necessary to respiration that it should be performed by means of a watery vehicle; the fish when brought on land becomes asphyxiated, not for want of air, but for want of water. Since the air is presented to it in a state, under which it cannot elaborate, and the evaporative effects of which it is unable to withstand. Its gill therefore quickly desiccates, and the animal perishes. The gill being in fact an unprotected lung, but the lung a protected gill; protected against, because exposed to evaporation, by a contrivance, which at the same time, by its mucous surfaces and secreting glands, ministers to its aqueous condition, for the terrestrial, like the aquatic, animal must be asphyxiated under a condition which deprives it of these means of extracting moisture.

"But how avails this digression, which I can fancy some pronouncing most irrelevant to the purpose. Man, say they, is not a fish, and what can the physiology of its respiration have to do with the subject under consideration? *Perhaps more than at first imagined, if, either for prevention or cure, the phthisical must advance a step towards the aqueous condition under which the fish respires.* For hereby, is understood a fact which has been brought to light by the experience of later years, that moisture is not prejudicial to the consumptive, as was at first supposed. Without going to the extent of saying, with some physicians of the present day, that ague, the abundant offspring of moisture, is antagonistic to phthisis, it may readily be conceived that a damp atmosphere, by presenting aspirable air to the lungs under a form more analogous to the fluid about to receive it, facilitates the changes which occur during respiration, changes—upon the due execution of which—depend the health of the individual.

"There is a very intimate connection between the vital energy of the creature and the activity of this function. Birds, some of which are destined for rapid and extraordinary exertions, possess, in comparison with other animals, large vesicular lungs, to which the bones, being hollow and containing air, are likewise auxiliary. How bold and energetic the movements of the hawk! how slow and indolent those of the turtle! On a smaller scale is witnessed in man a remarkable concord between the vigorous performance of this function and the robust health of the individual." P. 18.

Equally rational is the description given of the digestive function. Will our readers believe that any man, who has honestly received a professional diploma, could pen such nonsense as this?

"If indeed digestion, more correctly termed assimilation, be allied to fermentation, which some believe to be the case, the extrication of carbonic acid and other gases is an accompaniment of that process, which would then seem to be absorbed by the walls of that viscus. Carbon is necessary, but in different quantities, for the elaboration of the tissues of both plants and animals." P. 22.

The "viscus" here alluded to, we suppose to be the stomach.

With such specimens of our author's anatomical and physiological knowledge, no one can be surprised at the stupid blunders which he perpetrates, when he proceeds to treat of matters more immediately appertaining to practice. When describing the signs afforded by auscultation, we are told that Avenbrugger pointed out the applicability of percussion to the elucidation of chest diseases, towards the latter part of the *seventeenth* century! But this is a very minor blemish. In the very next page, we learn from the author himself the true why and wherefore of his appearing in print:

"It is not the object of this brief treatise to enter at any length into an exposition of the signs afforded by percussion and auscultation, because such details are only befitting a work intended solely for the professional eye, and also because the self-application to the detection of disease is impossible; and even were it possible, would be absurd and dangerous. Be it my endeavour to attempt a reasonable explanation of their phenomena, and so to recommend their general adoption, as a great step towards improvement in the treatment of this disease." P. 49.

Yet this is a work dedicated by permission to George J. Guthrie, Esq., F.R.S., F.L.S., late President of the Royal College of Surgeons of England, &c. &c. Doubtless Mr. Guthrie was utterly unaware of the character of the publication to which he has lent the sanction of his respectable name; and our only reason for alluding to this subject is to shew the necessity of men of repute in our profession not accepting every trumpery dedication that is offered to, or thrust upon, them. If sufficient evidence has not already been adduced to shew the nature of Dr. Deshon's production, what thinks the reader of the following?

"Need I tell you what happens every day? Hundreds of young females evidencing all the several symptoms of incipient phthisis, but which may merely denote mal-nutrition, and a proclivity to it, (for I defy any man to decide between them without the assistance of auscultation and percussion) are actually hurried into its confirmed phases by injudicious treatment—by purging, sweating, and cooling medicines; with cupping, leeches, and blistering; 'for look!' says the symptomatic physician, 'there is cough, difficult breathing, local pain, and bloody expectoration; actually hæmorrhage! We must subdue the inflammation.'" P. 59.

One of these "young females" is thus most interestingly described:

"But the state of the chrysalis being at an end, a new field is open for Mademoiselle to range in. She makes her début in society; she may be wondrously delicate and beautiful, and like Sylvia, all the world may admire her; she may be

'Like all that poets fancy, like all that lovers dream,'

but still possessed of an acquired or hereditary susceptibility of constitution, which soon becomes augmented by various descriptions of excitement. The ball, the theatre, late dinners, midnight suppers, form the category of her sphere of action. Her exhausted frame, after exposure to the chilling damp of a nocturnal air, seeks on a soft, luxurious bed, repose; but a feverish sleep, amidst the freshest hours of morning, is her fate." P. 75.

Among other remedies, Dr. Deshon mentions inhalation as a valuable agent in the treatment of phthisis. "Among its advocates," says he, "in more modern times, appear the names of Laennec, Drs. Copland, Elliotson, Corrigan, Willson, *Ramadge*, Hastings, and *Maddock*." The name of Scudamore is not so much as hinted at! By-the-bye, we doubt much whether our friend Dr. Hastings ever occupied so strange a position as that here accorded to him, between two such honourable worthies. Dr. Ramadge, we may mention, is repeatedly quoted by our author as a most esteemed authority; and, on one occasion, he appeals to the testimony of another notorious writer, who has declared that, "with quinine and other *chronothermal* remedies, he has cured or arrested at least 500 cases of consumption, many of them, too, apparently in very advanced stages." The *modus operandi* of inhalation—nothing but the vapour of tepid water, to which a piece of camphor may be added, is used—is thus described:—

"The benefits resulting from the inhalation of watery vapour, at the highest temperature that can comfortably be borne, which would range from 160° to 180° Farenheit, is two-fold. By entering and expanding the circumjacent pulmonary vesicles, a most salutary pressure is exercised upon the walls of the excavation, and the more or less partial or complete approximation of its sides is effected: while, at the same time, the faculty of aspiration and expiration of the sound air-cells being augmented, a state of the lungs is induced unfavourable to the further development of the disease.

"But this is not all, for the direct application of heat and moisture to the ulcerated cavity has a most soothing and beneficial influence upon the nerves and vessels of the part; alleviating, in a remarkable manner, morbid irritation, and increasing the circulation to the highest pitch consonant with health, favouring to the utmost, reparation of the pulmonary lesion." P. 140.

As a matter of course, Dr. Deshon can quote cases in illustration of the success of his practice. Sir Charles Scudamore will perceive, from the following narrative, that tubercular excavations can be cured without his favourite Iodine and Conium drops.

"Within the last twelve months a person sought my advice upon whom the good effects of inhalation, in healing a cavern, were so evident as to justify my introducing the case here. This patient manifested great debility and emaciation, and had night perspirations and profuse expectoration; he also suffered much from pains in the chest and larynx, which I expected to be ulcerated, to which nothing was so soothing as inhalation at a high temperature. Auscultation elicited pectoriloquy, and at once determined the nature of the case, which, indeed, was so marked, that a friend, who visited the patient for me, thought every hope of recovery forlorn, and advised a removal to his relatives and native air; yet this individual recovered, and now follows his usual avocations. It is not to be denied that inhalation, aided by appropriate medicines, saved this man's life, and that his continued health devolves upon his living judiciously, and using cold affusion and friction, to dispel occasional congestive pains which threaten, and would otherwise distress him." P. 141.

A most satisfactory and convincing case indeed! And now we have done with our painful and humiliating task. Would that it might with fairness have been avoided! But such cannot be, if the dignity and honour of the medical profession are to be upheld; for, assuredly, in no way is its character more signally tarnished than when any of its members are allowed to escape public censure and rebuke, when they bring discredit on its rightful claims. And here we are constrained to confess that there has been much in the medical literature of recent years, that is utterly inconsistent with the requirements not only of good taste and honourable feeling, but even of common truth and honesty. Scarcely a day passes over our heads but we meet with some flagrant example of the ill-directed love of public notoriety over-mastering the plain dictates of these too-much neglected guides. Numerous are the instances that might be quoted in proof of this charge. When we find a Fellow of the Royal Society lending himself to this sorry game, and condescending, for example, to borrow from the pages of the *Court Journal* a recommendatory notice of his work on a subject connected with the toilette graces of the person, what may we not expect from others who are more in the shade?

